# Supplementary material for: Single-Cell RNA Sequencing Reveals Multiple Pathways and the Tumor Microenvironment Could Lead to Chemotherapy Resistance in Cervical Cancer
Source: Front Oncol. 2021 Nov 26;11:753386. doi: 10.3389/fonc.2021.753386 (PMC8662819; doi:10.3389/fonc.2021.753386)
Supplement: Supplementary file 2 [file DataSheet_2.zip › Supplemental Material-Table S1.pdf]

Table S1. Summary metrics for 10x Genomics scRNA-seq barcoding and sequencing of five samples

	S1	S2	S3	S4	S5
**Estimated Number of Cells**	3282	5210	6425	6516	5679
**Mean Reads per Cell**	89,290	52,512	60,046	44,361	58,521
**Median Genes per Cell**	1,303	1,891	2,214	1,756	1,921
**Number of Reads**	293,049,455	273,586,827	385,792,793	289,059,207	321,987,201
**Valid Barcodes**	97.7%	97.6%	97.6%	98.7%	98.1%
**Sequencing Saturation**	79.8%	64.4%	65.5%	42.8%	68.6%
**Q30 Bases in Barcode**	96.1%	96.1%	95.9%	95.5%	96.1%
**Q30 Bases in RNA Read**	93.2%	93.1%	92.6%	92.6%	92.8%
**Q30 Bases in UMI**	95.8%	95.8%	95.6%	92.7%	95.7%
**Reads Mapped to Genome**	96.7%	96.9%	96.4%	96.4%	96.4%
**Fraction Reads in Cells**	88.8%	91.4%	86.1%	89.8%	90.4%
**Total Genes Detected**	21,855	22,928	24,211	23,681	24,310
**Median UMI Counts per Cell**	3,319	6,456	7,922	7,742	6,831
