# Supplementary material for: Single-Cell RNA Sequencing Reveals Multiple Pathways and the Tumor Microenvironment Could Lead to Chemotherapy Resistance in Cervical Cancer
Source: Front Oncol. 2021 Nov 26;11:753386. doi: 10.3389/fonc.2021.753386 (PMC8662819; doi:10.3389/fonc.2021.753386)
Supplement: Supplementary file 2 [file DataSheet_2.zip › Supplemental Material-Table S2.pdf]

Table S2. Enriched functions of differentially expressed genes (DEGs) in epithelial cells subpopulations

Description	GeneRatio	pvalue
Pathways of neurodegeneration - multiple	247/3157	2.7E-09
Amyotrophic lateral sclerosis	215/3157	2.48E-15
Alzheimer disease	197/3157	8.41E-09
Huntington disease	180/3157	8.71E-13
Parkinson disease	157/3157	7.12E-15
Prion disease	154/3157	2.83E-09
Human papillomavirus infection	151/3157	7.73E-03
Endocytosis	149/3157	5.04E-11
Herpes simplex virus 1 infection	147/3157	1E+00
Shigellosis	144/3157	2.91E-10
Salmonella infection	141/3157	9.56E-09
PI3K-Akt signaling pathway	133/3157	7.4E-01
MAPK signaling pathway	132/3157	2.19E-02
Thermogenesis	129/3157	1.24E-07
Coronavirus disease - COVID-19	127/3157	6.85E-07
Protein processing in endoplasmic	122/3157	5.62E-18
Epstein-Barr virus infection	117/3157	3.2E-08
Human T-cell leukemia virus 1 infection	117/3157	9.01E-06
Viral carcinogenesis	115/3157	3.09E-07
Ribosome	114/3157	1.76E-17
Pathogenic Escherichia coli infection	112/3157	2.43E-07
Human cytomegalovirus infection	112/3157	6.05E-04
RNA transport	108/3157	9.07E-08
Proteoglycans in cancer	107/3157	7.75E-05
