# Supplementary material for: Single-Cell RNA Sequencing Reveals Multiple Pathways and the Tumor Microenvironment Could Lead to Chemotherapy Resistance in Cervical Cancer
Source: Front Oncol. 2021 Nov 26;11:753386. doi: 10.3389/fonc.2021.753386 (PMC8662819; doi:10.3389/fonc.2021.753386)
Supplement: Supplementary file 2 [file DataSheet_2.zip › Supplemental Material-Table S3.pdf]

Table S3. Enriched functions of differentially expressed genes (DEGs) in B cells subpopulations

**Description**	**GeneRatio**	**pvalue**
Coronavirus disease - COVID-19	96/1141	4.42E-25
Pathways of neurodegeneration - multiple	93/1141	4.49E-04
Ribosome	90/1141	5.07E-37
Amyotrophic lateral sclerosis	81/1141	1.18E-05
Endocytosis	72/1141	1.06E-09
MAPK signaling pathway	71/1141	2.06E-06
Alzheimer disease	71/1141	3.3E-03
Salmonella infection	69/1141	9.68E-09
Shigellosis	68/1141	1.39E-08
Epstein-Barr virus infection	63/1141	2.48E-10
Prion disease	62/1141	6.75E-05
Huntington disease	62/1141	1.68E-03
Herpes simplex virus 1 infection	62/1141	8.8E-01
Human immunodeficiency virus 1 infection	59/1141	9.88E-08
Human T-cell leukemia virus 1 infection	59/1141	3.38E-07
Human cytomegalovirus infection	57/1141	4.48E-06
Parkinson disease	56/1141	1.97E-04
Human papillomavirus infection	55/1141	1.07E-01
Pathogenic Escherichia coli infection	54/1141	5.88E-07
Kaposi sarcoma-associated herpesvirus	52/1141	1.68E-06
Protein processing in endoplasmic	50/1141	1.8E-07
Viral carcinogenesis	50/1141	4.43E-05
PI3K-Akt signaling pathway	50/1141	5.23E-01
Ubiquitin mediated proteolysis	46/1141	1.11E-08
