# Supplementary material for: Single-Cell RNA Sequencing Reveals Multiple Pathways and the Tumor Microenvironment Could Lead to Chemotherapy Resistance in Cervical Cancer
Source: Front Oncol. 2021 Nov 26;11:753386. doi: 10.3389/fonc.2021.753386 (PMC8662819; doi:10.3389/fonc.2021.753386)
Supplement: Supplementary file 2 [file DataSheet_2.zip › Supplemental Material-Table S4.pdf]

Table S4. Enriched functions of differentially expressed genes (DEGs) in T cells subpopulations

Description	GeneRatio	pvalue
Pathways of neurodegeneration - multiple	138/1399	4.63E-11
Amyotrophic lateral sclerosis	127/1399	9.72E-17
Alzheimer disease	116/1399	7.89E-12
Coronavirus disease - COVID-19	108/1399	1.11E-25
Prion disease	102/1399	6.98E-16
Huntington disease	102/1399	3.17E-12
Parkinson disease	96/1399	4.92E-16
Endocytosis	93/1399	3.4E-14
Shigellosis	90/1399	1.62E-13
Salmonella infection	90/1399	3.66E-13
Ribosome	83/1399	2.32E-24
Human T-cell leukemia virus 1 infection	79/1399	1.19E-11
Human immunodeficiency virus 1 infection	77/1399	1.48E-11
Epstein-Barr virus infection	76/1399	2.65E-12
Thermogenesis	75/1399	9.65E-09
Human papillomavirus infection	75/1399	6.57E-03
MAPK signaling pathway	74/1399	3.41E-04
Human cytomegalovirus infection	72/1399	3.76E-08
Protein processing in endoplasmic	69/1399	5.79E-13
Viral carcinogenesis	68/1399	1.46E-08
Regulation of actin cytoskeleton	67/1399	6.04E-07
PI3K-Akt signaling pathway	66/1399	2.69E-01
Non-alcoholic fatty liver disease	64/1399	1.94E-13
Kaposi sarcoma-associated herpesvirus	64/1399	4.88E-08
